# Editorial: Renal Function in Acute and Chronic Kidney Diseases

**DOI:** 10.3389/fphys.2020.625353

**Published:** 2020-12-03

**Authors:** John D. Imig, Md Abdul Hye Khan, Xueying Zhao

**Affiliations:** ^1^Drug Discovery Center and Cardiovascular Center, Medical College of Wisconsin, Milwaukee, WI, United States; ^2^Department of Physiology, Morehouse School of Medicine, Atlanta, GA, United States

**Keywords:** diabetic nephropathy, acute kidney injury, hypertension, renal epithelium, glomerulus

## Introduction

Acute and chronic kidney diseases have devastating consequences on human health. Renal vascular function, glomerular filtration, and epithelial transport are required for water and electrolyte homeostasis. Nephrotoxicity and diseases such as hypertension, diabetes, and metabolic syndrome contribute significantly to acute and chronic kidney diseases (Barnett and Cummings, 2018). These pathological states impact on renal vascular and epithelial function and the ability for the kidney to maintain water and electrolyte homeostasis. Experimental and clinical studies over the past decade determined that there is a transition from acute to chronic kidney injury (Fiorentino et al., 2018). Acute kidney injury in the clinical setting can cause sustained alterations in epithelial transport and renal hemodynamics that increase the risk for developing chronic kidney disease (Sharfuddin and Molitoris, 2011). Currently, there are extremely limited options to treat acute and chronic kidney diseases.

A Research Topic on acute and chronic kidney diseases is timely because this is a very fast-moving field that is focused on the tremendous need for kidney disease therapeutics. Articles in this Research Topic advance our understanding of organismal, cellular, and molecular mechanisms that contribute to acute and chronic kidney diseases. The Research Topic has broad interest since it covers acute kidney injury, chronic kidney disease, diabetic nephropathy, and hypertension mediated kidney disease. Research articles span cell signaling, animal studies, human and animal disease pathology studies, renal hemodynamics, glomerular filtration, and renal epithelial transport studies. Thirty contributions focus on novel developments defining renal vascular and epithelial mechanisms that contribute to acute and chronic kidney diseases.

## Reviews

The Research Topic contains five review articles that discuss issues associated with acute and chronic kidney diseases. Macrophages and acute kidney disease are the focus of one review (Baek). Contributions to acute kidney injury for M1 pathogen destroying and inflammatory macrophages and the M2 immunosuppressive and tissue regeneration macrophages are discussed with an emphasis on the potential for therapeutic modulation (Baek). Mitochondrial fatty oxidation in acute and chronic kidney diseases that elicits tubular injury, inflammation, and fibrosis are explored in another review article (Jang et al.). Insight is provided on adenosine triphosphate (ATP) depletion, lipotoxicity, and mitochondrial energy metabolism in kidney diseases with a prospective for developing therapeutics. The theme of lipotoxicity and fibrosis in kidney diseases continues in a review article that focuses on sterol regulatory-element binding proteins (SREBPs) as mediators of kidney fibrosis via lipid-independent pathways (Dorotea et al.). Next, a mini-review examined the therapeutic potential for erythropoietin to combat renal fibrosis and chronic kidney disease (Zhang, Zhu et al.). Lastly, an opinion piece described heat strain, external workload, and chronic kidney disease in endurance athletes based in the tropical regions (Rojas-Valverde et al.). The authors advocate for more research for endurance athletes in the tropical regions to prevent chronic kidney disease in this population (Rojas-Valverde et al.). These reviews, mini-review, and opinion piece provide valuable insight on the pathology of acute kidney injury and chronic kidney disease, as well as, providing possible therapies to combat kidney diseases.

## Acute Kidney Injury

Mechanisms of acute kidney injury and potential therapeutic approaches were the emphasis of eight scientific articles in the Research Topic. The therapeutic potential for resolvin D1 to attenuate ischemia/reperfusion injury via the anaplastic lymphoma kinase/formyl-peptide receptor-2 (ALX/FPR2) pathway was demonstrated (Luan et al.). This article convincing evidence is presented that resolvin D1 protects against ischemia/reperfusion acute kidney injury by increasing regulatory T cell (Tregs) to reduce inflammation and renal tubular injury in mice (Luan et al.). Another therapeutic article described neferine, an alkaloid of the green seed of Lotus, on ischemia/reperfusion and lipopolysaccharide (LPS) induced acute kidney injury in mice (Li et al.). Pretreatment of neferine attenuated apoptosis and inflammation and increased kidney Klotho expression to mitigate acute kidney injury (Li et al.). Next, opioid regulation of rat renal epithelial cells exposed to hypoxia was evaluated (Luo et al.). This article found that δ-opioid receptor activation or inhibition regulates anti-inflammatory cytokines in renal epithelial cells exposed to hypoxia (Luo et al.). Anti-inflammatory cytokines were also the focus of a study in mice that had albumin overload induced acute kidney injury (Peruchetti et al.). The findings of this experimental study revealed that the interleukin-4 (IL-4) receptor α chain protects the kidney for albumin overload injury through modulation of the pro-inflammatory response (Peruchetti et al.). The acute kidney injury therapeutic theme continues with a study that demonstrates dexmedetomidine protects against LPS kidney injury in rats (Zhao et al.). Dexmedetomidine enhances kidney autophagy via the phosphoinositide 3-kinase/protein kinase B/mammalian target of rapamycin (PI3K/AKT/mTOR) pathway to decrease LPS induced renal damage (Zhao et al.). The attention on LPS renal injury in mice continued with an article on 15-hydroxyprostaglandin dehydrogenase (15-PGDH) treatment (Miao et al.). Inhibition of 15-PGDH with SW033291 decreased renal apoptosis, enhanced autophagy, and decreased oxidative stress rather than decreasing inflammation to reduce LPS induced acute kidney injury (Miao et al.). The last two acute kidney injury therapeutic articles utilized cisplatin-induced nephrotoxicity. The antioxidant, ferrerol was found to attenuate cisplatin induced kidney injury in mice through inhibiting reactive oxygen species-mediated oxidation, reducing inflammation, and decreasing apoptosis signaling pathways (Ma et al.). Another cisplatin-induced acute kidney injury study revealed that PTEN-induced kinase-1 (PINK1) knockout rats had ameliorated kidney damage via effects on mitochondrial fission and mitophagy (Zhou et al.). Taken together, this cadre of acute kidney injury articles provide several therapeutic targets and prospective drugs that could eventually improve outcomes in humans with acute kidney injury.

## Diabetic Nephropathy

A trio of studies evaluated kidney damage associated with diabetes. The first of these experimental studies evaluated resveratrol in rabbits with diabetic nephropathy injected with the contrast dye, iohexol, to induce acute kidney injury (Wang Y. et al.). Resveratrol protected against renal injury by activating the sirtuin-1/peroxisome proliferator-activated receptor gamma coactivator 1-α/hypoxia inducible factor 1-α (SIRT1/PGC-1α/HIF-1α) signaling pathway to reduce hypoxia, mitochondrial dysfunction, and tubular cell apoptosis (Wang Y. et al.). Next, the therapeutic potential for trimetazidine in streptozotocin-induced diabetic nephropathy was evaluated (Yang et al.). Trimetazidine prevented diabetic kidney damage through anti-fibrotic and anti-oxidative stress mechanisms (Yang et al.). The last diabetic nephropathy study utilized renal tubular epithelial cells and glomerular mesangial cells in culture that were exposed to high glucose (Wang L. et al.). Metabolomics of renal epithelial and mesangial cells exposed to high glucose were compared to human plasma samples from diabetic nephropathy patients to reveal potential biomarkers for diabetic nephropathy (Wang L. et al.). Future studies are needed to further test the therapeutic approaches and validate biomarkers for diabetic nephropathy.

## Chronic Kidney Disease

Four articles examining four different types of chronic kidney diseases are presented that tackle very different pathological aspects of chronic kidney disease. The first article tackles refining the mouse subtotal nephrectomy model to get consistent changes in chronic kidney disease (O'Sullivan et al.). Subtotal nephrectomy in male 129S2/SV mice resulted in progressive proteinuric renal disease, renal inflammation, and cardiac hypertrophy that was reliably reproducible (O'Sullivan et al.). Folic acid nephropathy and progressive fibrosis in mice was examined in response to Smad3 and c-Jun N-terminal kinase (JNK) pathway blockade (Jiang et al.). The Smad3 inhibitor, SIS3, and the JNK inhibitor, SP600125, given combined ameliorated progressive renal fibrosis through actions on PGC-1α (Jiang et al.). Bilateral ureteral obstruction and release in mice was used to determine the effects of the renin inhibitor, aliskiren (Hu et al.). This study found that aliskiren increased renal aquaporin 2 expression and suppressed NLR family pyrin domain containing 3 (NLPR3) inflammasome activation to improve the urinary concentration defect in ureteral obstruction and release (Hu et al.). Nephrogenesis disturbances induced by angiotensin type 1 (AT1) receptor blockade were the focus of the last chronic kidney disease article (Deluque et al.). Calcitriol treatment attenuated the AT1 receptor blockade altered renal microvascular differentiation (Deluque et al.). Overall, these four articles provide a snapshot of the wide variation in disease models and mechanisms that contribute to chronic kidney disease.

## Hypertension

Five articles in the Research Topic addressed hypertension and kidney disease. Hypertension and kidney disease in the Ren-2 transgenic rats focused on diuretic treatment (Vaněčková et al.). This study found that progressive proteinuria was lowered by combined AT1 receptor and endothelin type A (ET_A_) receptor blockade that was further reduced by the diuretic, hydrochlorothiazide (Vaněčková et al.). Endothelin-1 (ET-1) regulation in human proximal tubule cells was the focus of an article investigating cellular proliferation (Douma et al.). This study identified a long, non-coding RNA (lncRNA) that is an antisense for the gene that encodes ET-1 (*EDN1-AS*) which increases renal epithelial cell ET-1 secretion (Douma et al.). In another article, salt-sensitive hypertension and renal glomerular mitochondrial function were evaluated (Domondon et al.). Glomerular mitochondria were structurally and functionally defective in salt-sensitive hypertension rats which contributes to glomerulosclerosis (Domondon et al.). Continuing with the glomerular theme, another article assessed preglomerular arterioles in spontaneously hypertensive rats (SHR) for myogenic constriction (Nademi et al.). The findings in this study revealed that enhanced myogenic constriction of preglomerular arterioles was due to thromboxane A2 synthesis (Nademi et al.). The fifth hypertension article focused on the electrogenic Na^+^:${\text{HCO}}_{3}^{-}$ cotransporter NBCe2 (*Slc4a5*) in the renal epithelial cells of the connecting tubules and collecting ducts to blood pressure regulation (Barbuskaite et al.). Genetic deletion of NBCe2 resulted in net base extrusion and hypertension; however, hypertension was not observed in connecting tubule and intercalated cell NBCe2 knockout mice (Barbuskaite et al.). On the whole, these hypertension and kidney disease studies provide exciting and novel mechanisms responsible for blood pressure regulation and kidney disease progression.

## Clinical Studies

A Research Topic on acute and chronic kidney diseases would not be complete without clinical studies that explore therapies. Treatment for contrast-induced acute kidney injury in hypertensive patients evaluated the calcium channel blocker amlodipine (Yin et al.). Amlodipine given prior to contrast exposure resulted in protecting a Chinese cohort of hypertensive patients from contrast-induced acute kidney injury and increased long-term survival (Yin et al.). Another article explored the risk for acute kidney injury in patients with urinary tract infections (Hsiao et al.). This study found that the urinary tract infection, urolithiasis, increased the risk for uroseptic shock and acute kidney injury (Hsiao et al.). The ability for an anti-erythropoietin antibody to predict the need for erythropoietin treatment in dialysis patients with end stage renal disease was the focus of another study (Zhang, Bian et al.). This study found that the anti-erythropoietin antibody combined with age and potassium levels prior to dialysis predicted the need and dose of erythropoietin required during maintenance dialysis (Zhang, Bian et al.). Dialysis was also the focus of a study that explored inflammation and oxidative stress (Gutiérrez-Prieto et al.). The findings of this study revealed that adults that undergo peritoneal dialysis have significant changes in inflammation, oxidative stress, and oxidative damage to DNA (Gutiérrez-Prieto et al.). The devastating disease, hepatorenal syndrome, was examined in the fifth clinical study (Abdel-Razik et al.). Type-1 hepatorenal syndrome patients with an early reduction in ET-1 to nitric oxide ratio had better outcomes when treated with terlipressin and albumin (Abdel-Razik et al.). These five clinical studies highlight advances in the treatment of acute and chronic kidney diseases and provide insight on the considerable demand for better therapies.

## Conclusion

The Research Topic Renal Function in Acute and Chronic Kidney Diseases demonstrates emerging areas of research and clinical studies. This collection of 30 articles demonstrates that inflammation, mitochondrial dysfunction, and oxidative stress that contribute to the development and progression of renal diseases. Novel mechanisms and potential therapeutic targets are identified for acute kidney injury, hypertension induced kidney disease, diabetic nephropathy, and chronic kidney disease. Clinical studies highlighted the need for better kidney disease biomarkers and therapeutic approaches to improve kidney disease outcomes. Taken together, this Research Topic features recent advances and provides future directions for understanding renal function and treating acute and chronic kidney diseases.

## Author Contributions

JI conceived the content and drafted the manuscript. JI, MH, and XZ revised and approved the final manuscript. All authors contributed to the article and approved the submitted version.

## Conflict of Interest

The authors declare that the research was conducted in the absence of any commercial or financial relationships that could be construed as a potential conflict of interest.
